# Rapid Clinical Progression and Its Correlates Among Acute HIV Infected Men Who Have Sex With Men in China： Findings From a 5-Year Multicenter Prospective Cohort Study

**DOI:** 10.3389/fimmu.2021.712802

**Published:** 2021-07-22

**Authors:** Jing Zhang, Xiao-jie Huang, Wei-ming Tang, Zhenxing Chu, Qinghai Hu, Jing Liu, Haibo Ding, Xiaoxu Han, Zining Zhang, Yong-jun Jiang, Wenqing Geng, Wei Xia, Junjie Xu, Hong Shang

**Affiliations:** ^1^ NHC Key Laboratory of AIDS Immunology (China Medical University), National Clinical Research Center for Laboratory Medicine, The First Affiliated Hospital of China Medical University, Shenyang, China; ^2^ Key Laboratory of AIDS Immunology, Chinese Academy of Medical Sciences, Shenyang, China; ^3^ Key Laboratory of AIDS Immunology of Liaoning Province, Shenyang, China; ^4^ Collaborative Innovation Center for Diagnosis and Treatment of Infectious Diseases, Hangzhou, China; ^5^ Beijing Youan Hospital, Capital Medical University, Beijing, China; ^6^ Dermatology Hospital, Southern Medical University, Guangzhou, China; ^7^ University of North Carolina Project-China, Guangzhou, China; ^8^ School of Medicine, The University of North Carolina at Chapel Hill, Chapel Hill, NC, United States

**Keywords:** HIV, Antiretroviral (ARV), MSM, acute infection, rapid progressor

## Abstract

**Background:**

In the “treat all” era, there are few data on the nature of HIV clinical progression in middle-income countries. The aim of the current study was to prospectively analyze the clinical progression of HIV and its indicators among men in China with acute HIV who have sex with men.

**Methods:**

From 2009–2014 a total of 400 men with acute HIV infection (AHI) were identified among 7,893 men who have sex with men *via* periodic pooled nucleic acid amplification testing, and they were assigned to an AHI prospective cohort in Beijing and Shenyang, China. Rapid progression was defined as two consecutive CD4^+^ T cell counts < 350/µL within 3–24 months post-infection. Kaplan−Meier and Cox-regression analyses were conducted to identify predictors of rapid progression.

**Results:**

Among 400 men with AHI 46.5% were rapid progressors, 35.1% reached rapid progressor status by 12 months post-infection, and 63.9% reached rapid progressor status by 24 months. Rapid progression was associated with herpes simplex-2 virus coinfection (adjusted hazard ratio [aHR] 1.7, 95% confidence interval [CI] 1.2–2.3], depression (aHR 1.9, 95% CI 1.5–2.6), baseline CD4^+^ T cell count < 500/μL (aHR 3.5, 95% CI 2.4–5.1), higher baseline HIV viral load (aHR 1.6, 95% CI 1.2–2.3), acute symptoms lasting ≥ 2 weeks (aHR 1.6, 95% CI 1.1–2.2), higher body mass index (aHR 0.9, 95% CI 0.9–1.0), higher HIV viral load (aHR 1.7, 95% CI 1.4–2.1), set point viral load at 3 months (aHR 2.0, 95% CI 1.6–2.5), each 100-cell/μL decrease in CD4^+^ T cell count at 3 months (aHR 2.2, 95% CI 1.9–2.5), and baseline routine blood tests including white blood cell count < 5.32, hemoglobin ≥ 151, mean corpuscular hemoglobin ≥ 30.5, hemoglobin concentration ≥ 342, mean platelet count ≥ 342, lymphocytes ≥ 1.98, and mixed cell count ≥ 0.4 (all *p* < 0.05).

**Conclusion:**

Almost half of the patients underwent rapid clinical progression within 2 years after HIV infection. A treat-all policy is necessary and should be strengthened globally. Rapid progression was correlated with herpes simplex-2 virus coinfection, depression, low CD4^+^ T cell counts, and high set point viral load in acute infection stage. Rapid progression can be identified *via* simple indicators such as body mass index and routine blood test parameters in low and middle-income countries.

## Introduction

A “treat all” policy was recommended by the World Health Organization in 2016 to achieve the global aim of eliminating AIDS by 2030 (1). Since then an increasing number of countries have adopted the policy. Notably however, treat-all policy adoption has occurred at a disproportionate pace across geographical settings and communities. Most high-income countries have made treat-all a national policy, and the life expectancy of their people living with HIV (PLWHIV) have has greatly improved (2, 3). In middle and low-income regions such as West Africa, approximately 40% of healthcare sites are still dealing with HIV without this policy (4). A comprehensive understanding of clinical HIV progression, particularly rapid progression, has significant implications with respect to therapeutic strategies for public health providers and the probability of survival of people living with HIV (PLWHIV) in middle and low-income countries where a treat-all policy is not in place.

HIV disease progression involves a complex interplay of genetic, immunologic, and virologic factors during the early phase of the infection that determine whether an HIV-infected individual will rapidly progress to AIDS or not (5, 6). There is marked heterogeneity in disease progression rates among infected individuals, who can be broadly categorized as rapid progressors (RPs), typical progressors (TPs) and long-term non-progression (LTNPs). An estimated 70%–80% of HIV-positive patients are TPs, 10%–15% are RPs, and < 5% are LTNPs (7, 8). A recent review indicated that active replication of HIV during the extended clinically latent period results in a severe reduction in CD4^+^ T cell count. Thus, it is intuitive to link the rapidity of disease progression with high viral load and indolent disruption of CD4^+^ T cells, and eventual collapse of the immune system (9–11). Apart from CD4^+^ T cells and HIV dynamics, physiological issues, sexually transmitted infections, and baseline clinical characteristics also need to be taken into consideration when trying to comprehensively understand disease progression in HIV populations in low and middle-income countries.

To obtain HIV progression data, observation is necessary from the acute infection stage. Longitudinal HIV clinical progression data from large samples of RPs has become harder to obtain however, since more and more countries have adopted a treat-all policy. More importantly, even in fully-resourced settings acute HIV infection (AHI) is commonly missed or diagnosed belatedly because of the absence of virus-specific diagnostic tests in routine clinical HIV screening, and the non-specific nature of symptoms (12).

The aim of the current study was to use a large longitudinal prospective cohort of patients with AHI to identify the proportion of RPs present and predictors of rapid progression, in a middle-income country before a treat-all policy was adopted there, to facilitate greater understanding of HIV management globally. China adopted a treat-all policy in 2016. The study cohort were collated and monitored in the years from 2009–2014, and consisted of men who have sex with men (MSM) in China. MSM accounted for one third of the annual national newly reported HIV/AIDS cases in China (13). Before the treat-all era the incidence of HIV/AIDS among MSM increased from 3.24/100 person-years during the years from 2005–2008 to 5.50/100 person-years during the years from 2012–2014 (14). Preliminary data from mainland China suggest that clinical progression occurs most rapidly in HIV-positive MSM, compared to HIV-positive heterosexuals, and intravenous drug users (15). In a prior study the rate of progression in Chinese MSM was also greater than that in MSM in Europe (16).

## Materials And Methods

### Study Population, Recruitment, and Follow-Up Assessments

Three sampling methods were used to recruit eligible MSM attending voluntary counseling and testing centers in Beijing and Shenyang, China from 2009–2014; respondent-driven-sampling, snowball sampling, and peer referral/facility-based conventional sampling. Participants were eligible if they were born male, aged ≥ 18 years, had engaged in anal intercourse with any male in the last year, were willing to complete at least 2 years of follow-up, and provided written informed consent. Eligible HIV antibody-negative subjects in the initial screening at baseline and during the follow-up survey were both further screened *via* pooled polymerase chain reaction for HIV RNA. All HIV-negative (both antibody and RNA) participants at baseline were invited to attend follow-up assessments at 2-month intervals at designated clinics. To minimize loss-to-follow-up, a friendly reminder regarding the date of next follow-up visit was given to each participant well ahead of time. All diagnosed cases of AHI were enrolled into the China Acute HIV Infection Cohort Alliance and were followed up. All follow-up data were included in analyses until the initiation of highly-active anti-retroviral therapy (HAART).

### Definition of AHI and Enrollment Criteria for AHI Cohort

**Figure 1:**
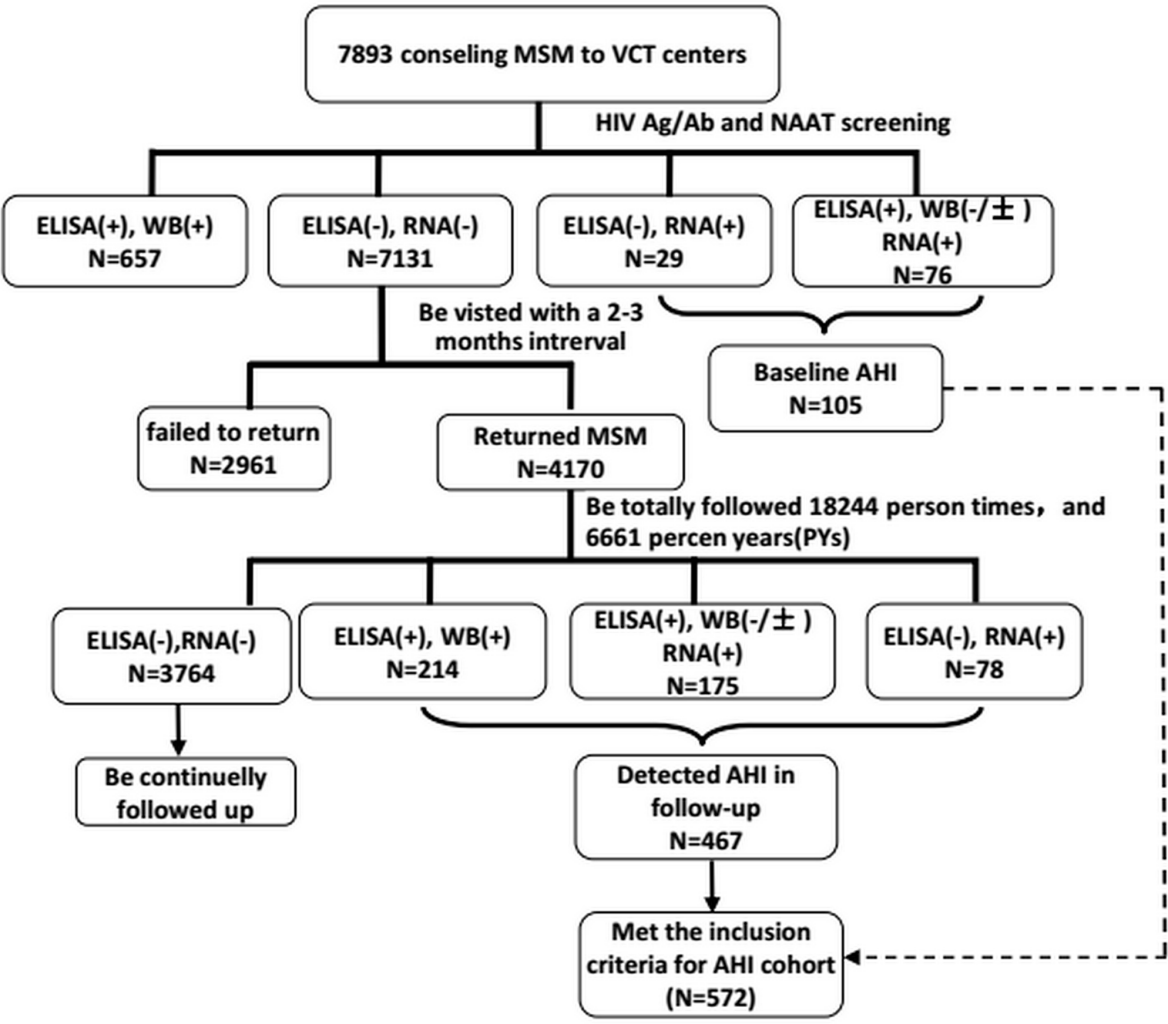
The flow chart of establishment of acute HIV infection cohort., 4170 subjects were followed 6661 person yeras (PYs) and be detected 467 HIV sereconverters, the HIV incidence rate HIV incidence density were 11.20%(95%CI, 10.26-12.20) and 7.01(95%CI, 6.41-7.65), respectively.

A participant was considered to have AHI if they (1) seroconverted during the follow-up period and the duration between the last antibody-negative test result and the first HIV-positive test was < 6 months (2), returned a negative HIV antibody test but a positive nucleic acid amplification test, or (3) the initial range of their HIV antibody testing was 0.5–1.0 OD and it subsequently increased within 2 weeks (17) (**Figure 1**). AHI cohort enrollment criteria were (a) AHI detected during follow-up of the HIV-negative MSM cohort, (b) written informed consent to attend prospective follow-up and HIV disease progression surveillance, and (c) AHI was followed up for > 120 days to determine HIV disease progression outcomes.

### Definition of Rapid Progressors

Participants were categorized as RPs if they returned ≥ 2 consecutive CD4^+^ T cell counts of < 350 cells/µL (with a gap of > 2 weeks between each test) during a 3–24-month period post-infection. Participants were categorized as non-rapid progressors (NRPs) if they only returned 1 CD4^+^ T cell count of < 350 cells/µL during a 3–24-month period post-infection, or they maintained CD4^+^ T cell counts of > 350 cells/µL (17).

### Measures

A face-to-face interview was conducted by a trained physician in a private room (to protect confidentiality) using a pre-tested interviewer-administered questionnaire to collect information on socio-demographic characteristics, sexual behaviors, and clinical symptoms. As per the standard guidelines the height and weight of each participant were also measured. At the end of the interview, pre-test counseling was provided and blood was collected from each participant for the required biological tests. During each follow-up visit, necessary biological, immunological, and virological tests were performed. HAART was initiated when the CD4^+^ T cell counts of HIV-seroconverted MSM reduced to < 350 cells/µL criteria. Notably, in May 2014 China updated its national anti-retroviral therapy (ART) guideline and recommended providing ART to PLWHIV with CD4^+^ T cell counts ≤ 500/µL, rather than ≤ 350/µL) (18).

### Depression

A 24-item Hamilton-Depression-Scale (19) was used to evaluate depression in patients with AHI during 4–6 months post-infection. Based on the total score, subjects were categorized as non-depressive (score < 7) or depressive (score ≥ 7).

### Testing for HIV and Other Sexually Transmitted Infections

A four-generation enzyme-linked immune adsorbent assay (ELISA) (Organon-Teknika-Boxtel Co. Ltd., Netherlands) was used to detect HIV antibodies at the initial screening. Positive samples were confirmed by HIV-1/2 western blotting (HIV Blot 2.2, Genelabs Diagnostics, Singapore). For detecting HIV-RNA, all ELISA-negative or western blot-negative/indeterminate samples were further assessed *via* nucleic acid amplification testing (COBAS AmpliPrep, COBAS TaqMan, Roche, Germany) using a 24-sample mini-pool strategy. ELISA-positive but western blot-negative/indeterminate test results were tested separately for HIV RNA, with a lowest detection limit of 20 copies/mL. To exclude false-positive determinations, nucleic acid amplification and western blotting tests were repeated for each sample identified as positive during the follow-up, until HIV antibody seroconversion events occurred subsequently.

Syphilis and herpes simplex virus-2 (HSV-2) were two of the most prevalent sexually transmitted infections (STIs) among MSM in China, and were highly correlated with HIV seroconversion. In previous studies the prevalence of syphilis among MSM in China was 9.1% (range 7.6%–10.8%) (20), and the mean prevalence of HSV-2 was 10.6% (range 6.2%–17.6%) (21). Therefore, all patients with AHI were screened for syphilis and HSV-2. Current syphilis infection was defined as testing positive *via* both the Rapid Plasma Reagin Test (Xinjiang Xindi, China) and the Treponema Pallidum Particle Agglutination Assay (Serodia Fujirebio Inc., Fuji, Japan). HSV-2 was tested using ELISA kits (HerpeSelect 2 ELISA IgG, Focus Technologies, Cypress, CA, USA) and seropositive patients were considered to be HSV-2 infected. Hepatitis was tested for using ELISA kits (Henan Huamei Bio-Engineering Co. Ltd., Shanghai Kehua Bio-Engineering Co. Ltd.) for detecting serum anti-hepatitis C virus core protein IgG antibodies and anti-hepatitis B surface antigen.

### Routine Blood Tests

Routine blood tests including hemoglobin (HGB), platelets (PLT), white blood cell (WBC) count, red blood cell (RBC) count, monocyte ratio (MONO%), neutrophil ratio (NEUT%), and monocyte count (MONO) were measured using a BC-6800 automatic blood analyzer (Mindray, China) whenever AHI subjects attended a followed-up visit.

### CD4^+^ T Cell Counts, HIV RNA Viral Load, and HIV Subtype Testing

CD4^+^ T cell counts were measured using a FACSCalibur flow cytometer (BD Bioscience, San Jose, CA, USA) based on a single-platform lyse-no-wash procedure using TruCOUNT tubes and TriTEST anti-CD4-FITC/CD8-PE/CD3-PerCP reagents (BD Bioscience). Viral load (VL) was detected using COBAS AmpliPrep-COBAS AMPLICOR HIV-1 MONITOR Test, version 2.0 (CAP-CA Roche Molecular Systems Inc.) with a detection limit of 20 copies/mL. HIV VL set point was calculated by taking average of all VL values measured during 4–6 months prior to the initiation of HAART. All VL and VL set point values were log_10_ transformed for data analysis. For HIV-1 subtyping, the pol sequences (entire protease and 256 codons of the RT sequence) were amplified and the edited sequences were aligned against the reference sequences available in the Los Alamos database.

### Statistical Analysis

Descriptive analyses were performed to determine and compare the distributions of demographic and behavioral factors, clinical correlates, STIs, and viral and immunological markers in RPs and NRPs at baseline. Mean, standard deviation (SD), median, and interquartile range (IQR) were used to describe continuous variables. Body mass index was calculated as body weight (kg)/height (m)^2^. Cox-regression analyses were performed to identify correlates of rapid progression with adjustment for socio-demographics, and adjusted hazard ratios (aHRs) and corresponding 95% confidence intervals (CIs) were calculated. Variables with *p* values < 0.20 in a bivariate model were included in a multivariate model. The Kaplan–Meier method was used to estimate time to reach end-points (CD4^+^ T cells < 500/µL, < 350/µL, and < 200/µL post-infection) and to determine the variation in rates among AHI cases with CD4^+^ T cells < 350/µL during the follow-up period across the strata of HIV genotypes/depression status/HSV-2 infection/VL at set point/duration of AHI symptoms. SAS version 9.3 was used for all statistical analyses. Graphs were prepared using SPSS 16.0 software. Midpoint between the last HIV-negative and the first HIV-positive antibody test or 14 days before HIV-RNA positive result were taken as possible time of infection (14).

### Ethics Statement

The Ethics Committee of The First Affiliated Hospital of China Medical University reviewed and approved the study protocol. Written informed consent was obtained from each participant before the study.

### Role of the Funding Source

The study sponsors had no role in study design, data collection, data analysis, interpretation of the data, writing the report, or the decision to submit the paper for publication.

## Results

### AHI Cohort

**Figure 2:**
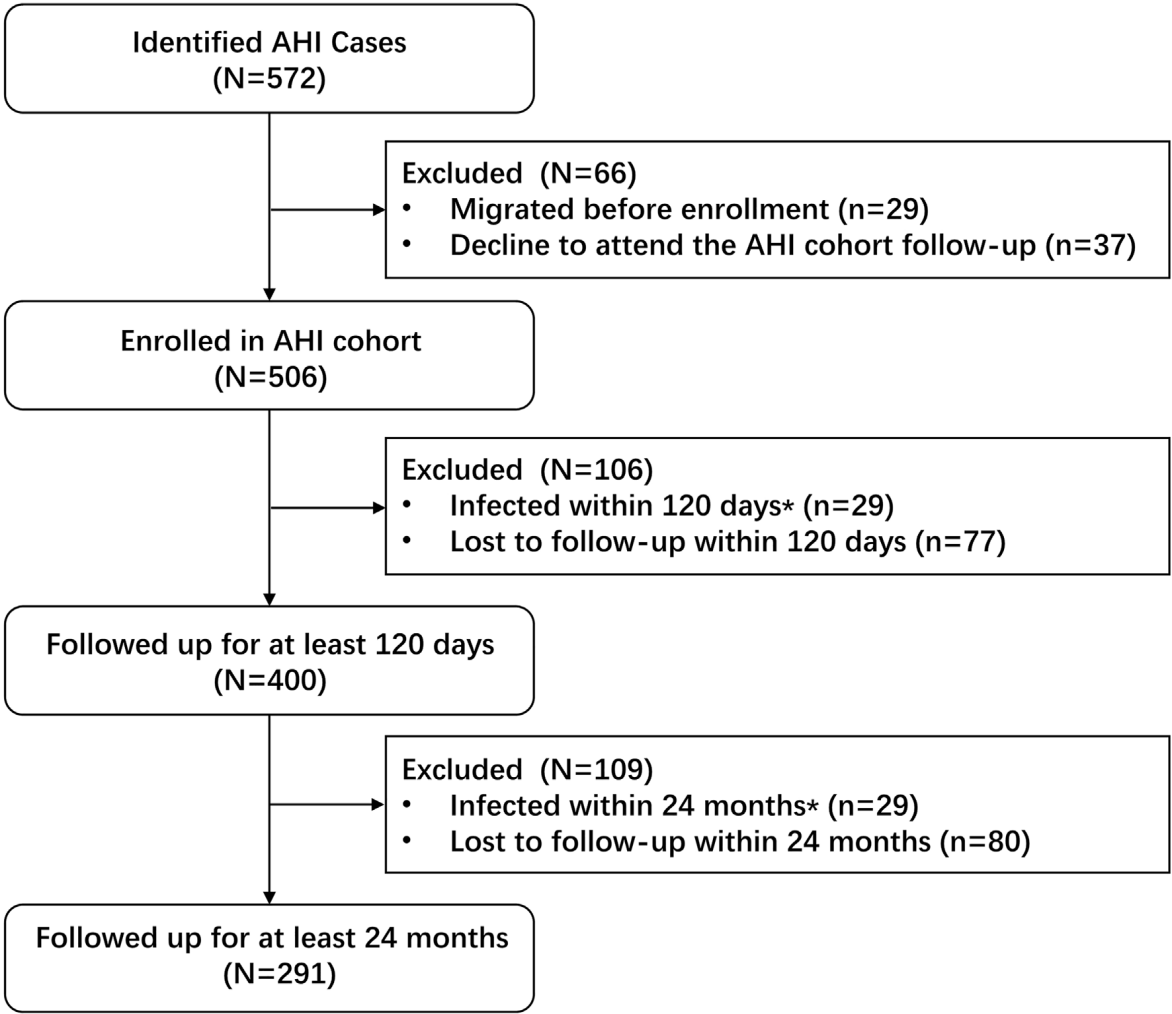
The diagram of AHI cohort subjects enrolment.

At the baseline, of 7893 consenting MSM, 105 newly diagnosed AHI cases were identified and 657 chronic cases (both ELISA-positive and western blot-positive) were excluded from the study. The remaining 7131 western blot and HIV-RNA-negative participants were invited for further follow-up assessments. Of 7131 MSM, 4170 (58.5%) returned to the study and 2961 (41.5%) were lost to follow-up. A total of 6661 person years of follow-up were observed for 4170 subjects with an HIV incidence density of 7.0 (95% CI 6.4–7.7) per 100 person years. Of the 4170 MSM, 467 were diagnosed with incident HIV infection during the study period, thus the total number of AHI cases was initially 572 (**Figure 1**). Of these, 400 who agreed to be followed up for > 120 days were included in the final analysis. The median viral load RNA set point of the 400 AHI cases was 4.6 (IQR 4.0–4.8) copies/mL. For the prediction of symptoms and events associated with CD4^+^ T cell counts < 350/μL, 291 patients with AHI who were followed up for at least 2 years were included in the Cox regression model (**Figure 2**).

### Sociodemographic and Clinical Characteristics at Baseline

Among a total of 400 patients with AHI the median age was 32 years (IQR 27–40), and the majority were of Han ethnicity (92.5%), had a monthly income > $350 US (52.5%), and had never been married (69.8%). One third (33.3%) had depression, and the mean body mass index was 20.3 kg/m^2^. More than 80% reported having < 3 male sex partners, and 46% had practiced both insertive and receptive sex in the last 3-months. Only 7.8% reported ever having had paid sex with a male partner. Syphilis seropositivity was detected in 119/400 (29.8%), 109/400 (27.3%) were positive for HSV-2, 24/400 (6%) were positive for hepatitis B surface antigen, and 13/400 (3.3%) were positive for anti-hepatitis C virus core protein IgG antibodies at baseline.

WBCs < 5 × 10^9^/L were detected in 156/400 (39.0%) patients with AHI, 218/400 (54.5%) had red cell counts < 5 × 10^9^/L, 207/400 (51.8%) had hemoglobin < 150 g/L, and 216/400 (54.0%) had platelet counts < 200 × 10^9^/L. The mean overall baseline CD4^+^ T cell count before HIV seroconversion was 752 cells/µL (SD 268.6). The mean initial CD4^+^ T cell count was 437 cells/µL (SD 195.6), and the mean log baseline HIV VL was 4.6 copies/mL (SD 1.1) after being diagnosed with AHI. Genetic analysis indicated that majority of patients had the CRF01_AE subtype (63.8%), followed by CRF07_BC (12.0%). During the first year post-seroconversion the mean CD4^+^ T cell count dropped by 32.1 cells/µL (IQR -78.5–151.7), whereas the mean annual drop was 79.3 cells/µL (IQR 2.8–151.2). The mean HIV VL was 4.3 copies/mL (SD 0.8). Approximately 62% of the patients had a history of acute symptoms, which on average lasted for < 2 weeks in 86% of cases. Fever was the most common symptom reported (49.5%), followed by malaise (31.2%).

During follow-up 186/400 (46.5%) patients with AHI were identified as RPs, and 186/400 (46.5%) were identified as NRPs. In comparisons of the baseline characteristics of RPs and NRPs, there were significant differences in age, monthly income, HSV-2 coinfection, depressive scores, acute infection symptoms, baseline routine blood test items, baseline CD4^+^ T cell count and VL, VL at set point, annual CD4^+^ T cell count reduction speed post-seroconversion, and mean log baseline HIV VL differed significantly (all *p* < 0.05). In the RP group there were greater proportions of patients aged > 30 years, monthly incomes < $350 US, HSV-2 coinfection, depression, duration of acute symptoms ≥ 2 weeks, duration of acute symptoms ≥ 3 weeks, and symptoms of acute malaise, and baseline HIV VL, annual CD4^+^ T cell count reduction speed post-seroconversion, set point VL were higher. Baseline CD4^+^ T cell counts were lower in the RP group. In routine blood test results WBC counts, RBC counts, platelets, lymphocyte counts, and eosinophil counts were higher in RPs than in NRPs, whereas the mean platelet volume was lower (**Table 1**).

**Table 1:** Demographics, behaviors, and acute symptoms in a cohort of patients with AHI (N = 400).

Characteristics	Categories/level	All (N = 400)	Non-rapid Progressors (*n* = 214)	Rapid progressors (*n* = 186)	*p* value
**Age (years)**	≤ 30	42.0% (168)	48.6% (104)	34.4% (64)	0.004
	> 30	58.0% (232)	51.4% (110)	65.6% (122)	
**Education level**	Junior high school and below	35.5% (142)	33.2% (71)	50.0% (71)	0.413
	Senior high school and below	29.0% (116)	28.5% (61)	29.6% (55)	
	College and above	35.5% (142)	38.3% (82)	32.3% (60)	
**Ethnicity**	Non-Han	7.5% (30)	7.9% (17)	7.0% (13)	0.718
	Han	92.5% (370)	92.1% (197)	93.0% (173)	
**Monthly income (US dollars)**	≤ 350	47.5% (190)	42.1% (90)	53.8% (100)	0.019
	> 350	52.5% (210)	57.9% (124)	46.2% (86)	
**Marital status**	Never married	69.8% (279)	72.9% (156)	66.1% (123)	0.142
	Ever/currently married	30.2% (121)	27.1% (58)	33.9% (63)	
**BMI (IQR)**		20.3 (19.6–20.8)	20.3 (19.9–21.3)	20.3 (19.3–20.5)	0.158
**Syphilis**	Positive	29.8% (119)	29.0% (62)	47.9% (57)	0.715
	Negative	70.3% (281)	71.0% (152)	52.1% (129)	
**HSV-2**	Positive	27.3% (109)	21.0% (45)	34.4% (64)	0.003
	Negative	72.8% (291)	79.0% (169)	65.6% (122)	
**HBsAg**	Positive	6.0% (24)	5.6% (12)	6.5% (12)	0.723
	Negative	94.0% (374)	94.4% (202)	93.5% (174)	
**Anti-HCV IgG antibodies**	Positive	3.3% (13)	2.8% (6)	3.8% (7)	0.589
	Negative	96.8% (387)	97.2% (208)	96.2% (179)	
**Baseline Routine blood tests**					
WBC (IQR)		5.3 (4.6-6.4)	5.5 (4.8–6.7)	5.1 (4.4–5.9)	< 0.001
RBC (IQR)		4.9 (4.7–5.3)	5.0 (4.7–5.3)	4.9 (4.6–5.2)	0.039
HGB (IQR)		151.0 (142.0–158.0)	152.0 (144.0–159.0)	150.0 (142.0–156.3)	0.070
HCT (IQR)		43.2 (39.0–45.9)	43.7 (39.1–46.1)	42.8 (37.9–45.5)	0.084
MCV (IQR)		89.3 (86.4–92.0)	89.3 (86.2–91.9)	89.4 (86.5–92.3)	0.886
MCH (IQR)		30.5 (29.3–31.4)	30.5 (29.2–31.5)	30.5 (29.5–31.4)	0.808
MCHC (IQR)		342.0 (329.0–350.0)	344.0 (330.8–351.0)	341.0 (328.8–350.0)	0.443
PLT (IQR)		195.0 (165.0–229.0)	200.5 (173.5–240.0)	187.5 (159.8–223.0)	0.002
LYM (IQR)		2.0 (1.6–2.5)	2.1 (1.7–2.7)	1.8 (1.4–2.4)	< 0.001
MXD (IQR)		0.4 (0.3–0.5)	0.4 (0.3–0.5)	0.4 (0.3–0.5)	0.067
NEUT (IQR)		2.9 (2.2–3.7)	2.9 (2.2–3.7)	2.7 (2.0–3.6)	0.082
PDW (IQR)		12.6 (11.5–14.4)	12.6 (11.2–14.1)	12.8 (11.9–14.6)	0.160
MPV (IQR)		10.3 (9.6–11.0)	10.1 (9.6–10.8)	10.5 (9.7–11.2)	0.002
PLCR (IQR)		25.3 (19.0–30.7)	25.3 (20.3–29.3)	25.9 (16.7–31.7)	0.156
EOS (IQR)		0.06 (0.03–0.11)	0.07 (0.04–0.13)	0.06 (0.03–0.09)	0.005
BAS (IQR)		0.02 (0.01–0.03)	0.02 (0.01–0.02)	0.02 (0.01–0.03)	0.075
**Depression^#^**	Yes	33.3% (133)	23.8% (51)	44.1% (82)	< 0.001
	No	66.7% (267)	76.2% (163)	55.9% (104)	
**Baseline CD4^+^ T cell counts (SD)**		437 (195.6)	521 (192)	350 (157.0)	< 0.001
**Log baseline HIV VL, copies/mL (SD)**		4.6 (1.1)	4.5 (1.1)	4.8 (1.0)	0.009
**First year CD4^+^ T cell counts (IQR)**		32.1 (-78.5–151.7)	10.0 (-95.2–145.3)	50.5 (-51.1–155.9)	0.155
**Annual CD4^+^ T cell counts (IQR)**		79.3 (2.8–151.2)	46.8 (-26.3–119.1)	109.6 (40.9–235.4)	< 0.001
**Setpoint VL log copies/mL (IQR)**		4.3 (0.8)	4.1 (0.9)	4.5 (0.7)	< 0.001
**History of acute infection symptoms**	Yes	61.8% (247)	57.9% (127)	64.5% (120)	0.289
	No	38.2% (153)	42.1% (87)	35.5% (66)	
**Duration of acute symptoms**	≥ 2 weeks	14.5% (58)	8.9% (19)	21.0% (39)	0.001
	< 2 weeks	85.5% (342)	91.1% (195)	79.0% (147)	
**Number of acute symptoms**	≥ 3	28.0% (112)	23.4% (50)	41.2% (62)	0.027
	< 3	72.0% (288)	76.6% (164)	58.8% (124)	
**Fever**	Yes	49.5% (198)	45.3% (97)	54.3% (101)	0.073
	No	50.5% (202)	54.7% (117)	45.7% (85)	
**Malaise**	Yes	31.2% (108)	24.8% (53)	29.6% (55)	0.039
	No	68.8% (238)	75.2% (145)	70.4% (93)	
**Lymphadenopathy**	Yes	8.5% (34)	6.1% (13)	11.3% (21)	0.062
	No	91.5% (366)	93.9% (201)	88.7% (165)	
**Sore throat**	Yes	26.0% (104)	22.4% (48)	30.1% (56)	0.081
	No	76.0% (296)	77.6% (166)	69.9% (130)	
**Diarrhea**	Yes	13.7% (54)	12.6% (27)	14.5% (27)	0.507
	No	86.3% (341)	87.4% (187)	85.5% (154)	
**Headache**	Yes	15.8% (43)	15.4% (33)	16.1% (30)	0.846
	No	84.2% (337)	84.6% (181)	83.9% (156)	
**Nausea or vomiting**	Yes	9.0% (36)	7.0% (15)	11.3% (21)	0.136
	No	91.0% (364)	93.0% (199)	88.7% (165)	
**Rash**	Yes	9.3% (37)	7.9% (17)	49.5% (20)	0.334
	No	90.7% (363)	92.1% (197)	50.5% (166)	

^#^Hamilton Anxiety Scale.

BMI, body mass index; WBC, white blood cell count; RBC, red blood cell count; HGB, hemoglobin; HCT, hematocrit; MCV, mean corpuscular volume; MCH, mean corpuscular hemoglobin; MCHC, mean corpuscular hemoglobin concentration; PLT, platelets; LYM, lymphocyte count; MXD, absolute value of intermediate cells; NEUT, neutrophil count; PDW, platelet distribution width; MPV, mean platelet volume; PLCR, platelet larger cell ratio; EOS, eosinophil count; BAS, basophil count.

### HIV/AIDS Progression During Follow-up

The median duration between the estimated date of HIV infection and AHI detection was 38 days (IQR 30–70), and the median follow-up duration post-infection was 421 days (IQR 187–716) (**Table 1**). ART was initiated in 82 cases with a CD4^+^ T cell count < 350/µL. The median duration between the estimated date of infection and ART initiation was 774 days (IQR 444–1020). The median time from the estimated HIV infection date to a positive results was 30 days (IQR 24–37), and the median time between an initial indeterminate western blot result and a positive result was 52 days (IQR 38–79).

**Figure 3:**
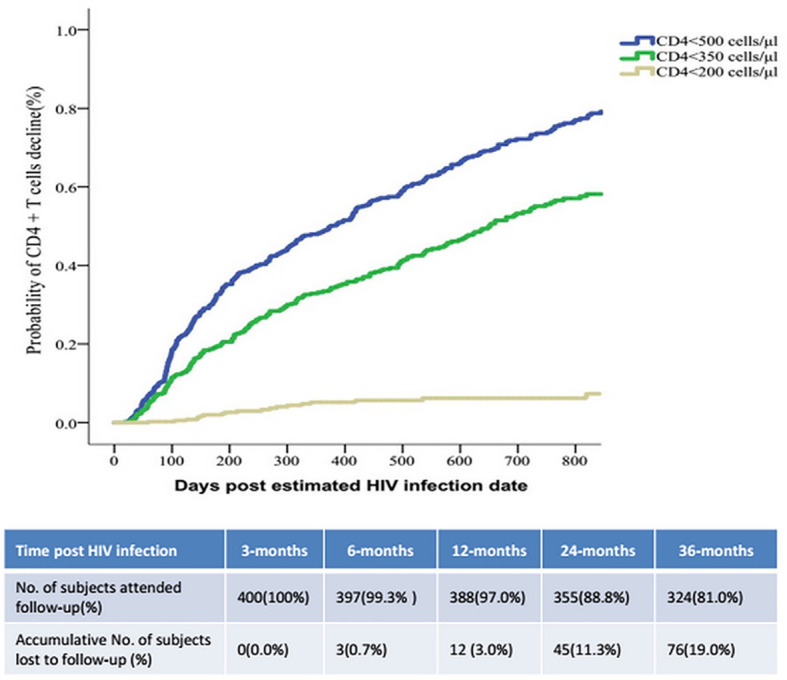
The speed of immunological progression events of CD4+T cell counts decreasing post HIV infection.

During follow-up 186/400 (46%) of the patients with AHI were identified as RPs (**Table 1**). The respective median numbers of days after the assumed HIV infection date until the occurrence of the endpoint events of CD4^+^ T cell count < 500/μL, < 350/μL, and < 200/μL were 234 (IQR 105–512), 269 (IQR 127–572), and 344 (IQR 144–760). Immunological progression to a CD4^+^ T cell count < 350/µL at 3, 6, 12, and 24 months occurred in 9.0%, 20.1%, 35.1%, and 63.9% of participants respectively. At the same timepoints 1.8%, 3.0%, 5.6%, and 11.3% of the participants exhibited CD4^+^ T cell count reductions to < 200 cells/µL, and 13.3%, 32.8%, 47.8%, and 67.8% exhibited CD4^+^ T cell count reductions to < 500 cells/µL (**Figure 3**).

The mean respective CD4^+^ T cell counts per µL were 380 ± 131, 343 ± 140, 305 ± 89, 296 ± 103, and 281 ± 62 at 90, 180, 360, 540, and 720 days post-infection in the RP group. These values were all significantly lower than the corresponding values in the NRP group at the same timepoints (588 ± 181, 552 ± 209, 532 ± 158, 521 ± 137, and 510 ± 168 cells/µL, all *p* < 0.001). During the first year after seroconversion, CD4^+^ T cell counts decreased, by a median of 32 cells/µL (IQR 79–152). The median overall annual decrease was 79 cells/µL (IQR 3–151).

### Correlates of Rapid Disease Progression

**Figure 4:**
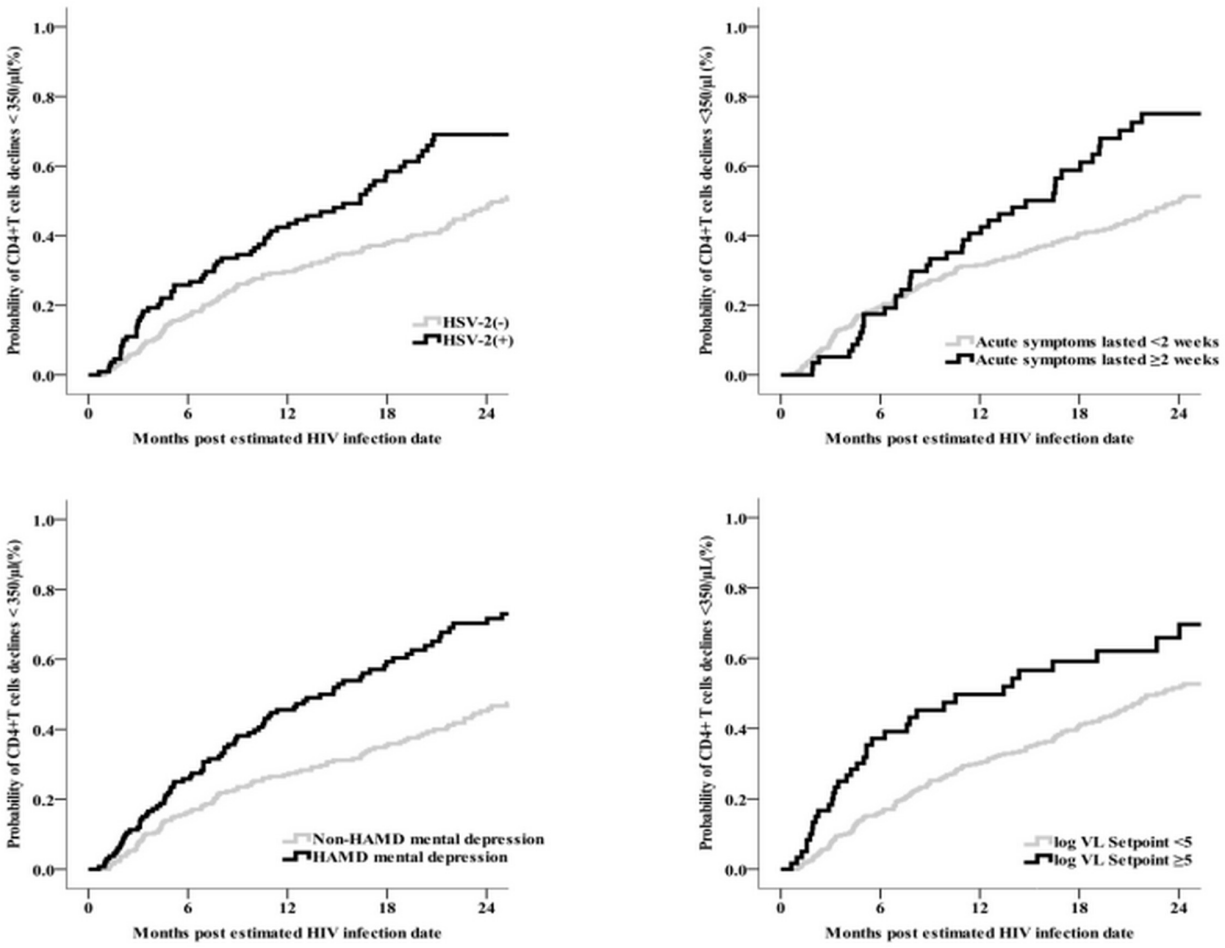
The relationship graphs of predictors of HSV-2, acute symptoms lasted time, depression and CD4<350/μl event (N = 400).

In Kaplan-Meier analysis participants with depression at baseline, HSV-2 coinfection at baseline, set point HIV VL ≥5 log_10_ copies/mL (*vs.* < 5), and persistence of symptoms ≥ 2 weeks (*vs.* < 2 weeks) reached the endpoint of a CD4^+^ T cell count < 350/μL at a significantly faster rate (**Figure 4**). In multivariable Cox regression models, baseline factors associated with rapid clinical progression included HSV-2 coinfection (aHR 1.7, 95% CI 1.2–2.3), depression (aHR 1.9, 95% CI 1.5–2.6), CD4^+^ T cell count < 500/μL (aHR 3.5, 95% CI 2.4–5.1), HIV VL > 5 log_10_ copies/mL (aHR 1.6, 95% CI 1.2–2.3), duration of acute symptoms ≥ 2 weeks (aHR 1.6, 95% CI 1.1–2.2), and baseline routine blood test items (WBCs < 5.3/L, hemoglobin < 151/L, mean corpuscular hemoglobin < 30.5 pg, mean corpuscular hemoglobin concentration < 342 g/L, platelets < 195/L, lymphocyte count < 2.0%, and absolute value of intermediate cells < 0.4%; all *p* < 0.05); higher body mass index (aHR 0.9, 95% CI 0.9–1.0) and mean platelet volume < 10.3 fL (aHR 0.6, 95% CI 0.5–0.9) were negatively associated with rapid progression (**Table 2**). HIV VL at 3 months (aHR 1.7, 95% CI 1.4–2.1, per log increase), set point HIV VL (aHR 2.0, 95% CI 1.6–2.5), and each 100-cell/μL decrease in CD4^+^ T cell count at 3 months (aHR 2.2, 95% CI 1.9–2.5) were associated with an increased risk of rapid progression.

**Table 2:** Factors associated with rapid HIV disease progression (CD4^+^ T cell count < 350/μL within 2 years of infection) according to Cox regression analysis (*n* = 291).

Sociodemographic characteristics	Category/level	Crude HR (95% CI)	*p* value	Adjusted HR (95% CI)	*p* value
**Medical history/comorbidities**					
Body mass index (IQR)		0.9 (0.9–1.0)	0.032	0.9 (0.9–1.0)	0.038
Depression	Positive	1.8 (1.4–2. 4)	< 0.001	1.9 (1.5–2.6)	< 0.001
Syphilis	Positive	1.1 (0.81–1.5)	0.566	1.1 (0.8–1.5)	0.597
Herpes simplex virus-2	Positive	1.6 (1.2–2.1)	< 0.004	1.7 (1.2–2.3)	0.001
Hepatitis B surface antigen	Positive	0.9 (0.5–1.7)	0.828	1.1 (0.6–2.0)	0.677
Anti-hepatitis core virus IgG antibodies	Positive	1.2 (0.6–2.5)	0.676	1.1 (0.5–2.4)	0.774
**Baseline routine blood tests** ^*^					
White blood cell count	< 5.3/L	1.5 (1.1–2.0)	0.007	1.5 (1.1–2.0)	0.006
Red blood cell count	< 4.9/L	1.3 (1.0–1.8)	0.047	1.3 (1.0–1.8)	0.046
Hemoglobin	< 151/L	1.5 (1.1–2.0)	0.007	1.5 (1.1–2.0)	0.008
Hematocrit	< 43.2%	1.0 (0.8–1.3)	0.991	1.0 (0.7–1.3)	0.965
Mean corpuscular volume	< 89.3 fL	0.9 (0.6–1.1)	0.279	0.8 (0.6–1.1)	0.233
Mean corpuscular hemoglobin	< 30.5 pg	1.5 (1.1–2.0)	0.008	1.5 (1.1–2.0)	0.005
Mean corpuscular hemoglobin concentration	< 342 g/L	2.1 (1.6–2.9)	< 0.001	2.2 (1.6–2.9)	< 0.001
Platelet count	< 195/L	1.6 (1.2–2.2)	0.001	1.7 (1.2–2.2)	0.001
Lymphocyte count	< 2.0%	1.4 (1.1–1.9)	0.013	1.4 (1.1–1.9)	0.022
Absolute value of intermediate cells	< 0.4%	1.4 (1.1–1.9)	0.017	1.4 (1.0–1.9)	0.030
Neutrophil count	< 2.9%	1.3 (1.0–1.7)	0.071	1.3 (1.0–1.8)	0.043
Mean platelet volume	< 10.3 fL	0.7 (0.5–0.9)	0.005	0.6 (0.5–0.9)	0.003
Eosinophil count	< 0.06%	0.8 (0.6–1.0)	0.097	0.8 (0.6–1.1)	0.115
Basophil count	< 0.02%	0.8 (0.6–1.0)	0.093	0.8 (0.6–1.0)	0.061
**Immunologic/virologic tests**					
Baseline HIV viral load > 5 log_10_ copies/mL		1.6 (1.2–2.2)	0.002	1.6 (1.2–2.3)	0.003
HIV viral load at 3 months (by 1 log_10_ copies/mL increase)		1.7 (1.4–2.1)	< 0.001	1.7 (1.4–2.1)	< 0.001
Set point HIV viral load (by log_10_ copies/mL increase)		2.0 (1.6–2.5)	< 0.001	2.0 (1.6–2.5)	< 0.001
Baseline CD4^+^ T cells < 500/µL		3.5 (2.4–5.1)	< 0.001	3.5 (2.4–5.1)	< 0.001
CD4^+^ T cell count at 3 months (by 100 cells/µL decrease)		2.2 (1.9–2.4)	< 0.001	2.2 (1.9–2.5)	< 0.001
**Acute symptoms**					
History of acute symptoms		1.1 (0.8–1.5)	0.637	1.1 (0.8–1.5)	0.519
Duration of acute symptoms	≥ 2 weeks	1.4 (1.0–1.9)	0.023	1.6 (1.1–2.2)	0.013
Number of acute symptoms	≥ 3	1.2 (0.9–1.7)	0.161	1.3 (1.0–1.8)	0.050
Fever		1.2 (0.9–1.6)	0.176	1.3 (1.0–1.7)	0.113
Malaise		1.2 (0.9–1.7)	0.310	1.3 (1.0–1.9)	0.101
Lymphadenopathy		1.6 (1.0–2.5)	0.052	1.6 (1.0–2.6)	0.032
Sore throat		1.2 (0.9–1.7)	0.235	1.3 (1.0–1.8)	0.067
Diarrhea		1.5 (1.0–2.3)	0.053	1.3 (0.9–1.9)	0.227
Headache		1.4 (1.0–1.9)	0.062	1.0 (0.7–1.5)	0.908
Nausea or vomiting		1.3 (0.8–2.0)	0.279	1.4 (0.9–2.2)	0.140
Rash		1.2 (0.7–1.9)	0.510	1.2 (0.8–2.0)	0.395

^*^Baseline routine blood test items were grouped according to medians.

CI, confidence interval; HR, hazard ratio; IQR, interquartile range; RP, rapid progression.

## Discussion

In this multicenter study of MSM in China before the “treat all” era, patients with AHI were prospectively followed up. Rapid progression occurred in 186/400 (46.5%) of the patients, and was more prevalent in those with HSV-2 coinfection, depression, low CD4^+^ T cell counts, and a high VL at baseline, and in those with a specific range of baseline routine blood test results. *Via* prospective follow-up identifying the nature of HIV disease progression in acutely infected MSM before the implementation of a treat-all policy, the current study demonstrates the urgency of early ART in middle and low-income countries. By combining epidemiology, clinical characteristics, immunology, and virology data, the study also quantified correlates of rapid disease progression other than genetic polymorphisms (22), rate of CD4^+^ T cell decline and HIV RNA change (16), and primary CXCR4 coreceptor tropism reported in previous studies (23, 24).

Almost half of the participants underwent rapid disease progression after HIV seroconversion. It is estimated that 70%–80% of HIV-positive individuals are typical progressors, 10%–15% are RPs, and < 5% are long-term NRPs (25). The proportion of men who experienced an immunological progression event (*i.e.*, CD4^+^ T cell count < 350 cells/µL) within 2 years post-infection in the present study was 63%, which is higher than that reported in studies conducted in South West Africa (55%) and South Africa (44%) (17, 26). The predominant HIV subtype varies across countries (CRF_AE in the current study, subtype C in South Africa) and this may have contributed to the differences observed in disease progression.

Consistent with the current study, several longer time follow-up studies have reported strong associations between depression and poor clinical outcomes such as AIDS symptoms or mortality in HIV-infected individuals (27, 28). The present study further verified that depression can accelerate the progression of the HIV disease at early infection stage. With regard to mechanisms underlying this association, it may be that adverse psychosocial factors stimulate the hypothalamic-pituitary-adrenal axis and sympathetic nervous system, leading to increased secretion of cortisol and catecholamine. These antecedent events probably affect disease progression in several ways such as increased HIV replication, enhanced expression of coreceptors, and altered innate and adaptive cellular mechanisms (28). MSM are still highly stigmatized in China and many middle and low-income countries, and mental health evaluation and treatment services are scarce in general HIV/AIDS clinics. Thus, screening for depression, management of stress, and providing friendly psychosocial interventions coupled with social support in HIV patients may improve adherence to treatment, and ultimately better survival.

In the present study concurrent HSV-2 infection was associated with rapid progression, though findings from previous studies are variable. It has been reported that HSV-2 coinfection increases the risk of HIV acquisition three-fold irrespective of sex, but the role of coinfection in disease progression remains unclear (29, 30). A recent HIV vaccine trial among MSM suggested that HIV/HSV-2 coinfection may influence disease progression by altering pro-inflammatory cytokines and enhancing viral replication, though the findings were not statistically significant (30). Incorporating HSV-2 screening and subsequent management into comprehensive HIV care may slow disease progression.

Associations between routine blood test results, particular hemoglobin, WBCs, and platelets, and the speed of disease progression in individuals with established HIV/AIDS have been reported (31–33). Notably however, in one prior study no such significant relationships were detected (17). The large sample size in the current study provided sufficient statistical power to confirm these associations, as well as enabling the identification of other routine blood test items (*i.e.*, mean corpuscular hemoglobin, mean corpuscular hemoglobin concentration, lymphocyte count, absolute value of intermediate cells, and mean platelet volume) that predicted rapid disease progression but have received little attention in previous studies. The use of these routine tests could facilitate the detection of RPs among patients with AHI without treat all policy, and assist healthcare professionals with decisions regarding ART initiation, especially in settings with limited resources for CD4^+^ T cell testing (33, 34).

In the present study individuals with a baseline CD4^+^ T cell count < 500/µL underwent rapid disease progression. This relationship between disease progression and CD4^+^ T cell counts is well-established (9–11). Prior research indicates that the likelihood of disease progression to AIDS increases significantly at < 350 cells/µL, and is highest at < 200 cells/µL (25). Interestingly, more rapid loss of CD4^+^ T cells has been reported in participants in the Beijing PRIMO cohort compared with those in Western countries (16). Disparate HIV subtypes, different host immune function, higher prevalence of coinfections, and differences in lifestyle likely contributed to such findings. Therefore, continuous screening for CD4^+^ T cell counts during follow-up visits in acutely infected MSM is crucial for timely initiation of treatment, monitoring the efficacy of anti-retroviral drugs, and disease prognosis. As in previous reports (35), in the current study participants with a higher baseline HIV VL and HIV VL at 3 months underwent more rapid disease progression than those with lower levels. A longer duration of acute symptoms was also associated with rapid progression, consistent with an earlier study (36).

The present study had some limitations. Convenience sampling was used to recruit MSM, thus extrapolation of results beyond the study should be done with caution. Although care was taken to avoid over representation of participants linked with large social networks, chances of bias may not have been completely eliminated. Given information on sexual behaviors was all self-reported, the extent of social desirability bias and influence of accuracy of memory on these responses remains questionable. Loss to follow-up was another major limitation of the study, and may have introduces some biases with respect to the analysis if characteristics and behaviors differed between those who were retained in the cohort and those who were lost to follow-up. Due to limited resources we only included syphilis and HSV-2 into the STI testing, though notably standard consultation and referral was provided for all participants with STI symptoms. Despite these limitations and the scarcity of data pertaining to determinants of rapid disease progression in MSM, by virtue of large sample size, robust methodology, advanced analyses, and long follow-up period, we believe that the current study generated important insights into the dynamics of HIV disease progression in male homosexuals.

The study results have particular significance for health in low and middle-income countries as they seek to implement and expand early HIV ART strategies, particularly among members of the MSM population. The accessibility and acceptability of ART by HIV-positive MSM is often limited in such settings. In China for example, approximately a third of HIV-positive MSM do not accept ART before their CD4^+^ T cell levels decrease to <350 cells/µL (37, 38). The prevalence of other infections such as tuberculosis, syphilis, hepatitis, and herpes may also exacerbate disease progression in these countries. Much work is still needed to control the HIV epidemic among MSM. The present study provides key evidence to support such efforts and help identify men at greatest risk of rapid disease progression.

## Data Availability Statement

The original contributions presented in the study can be obtained from the corresponding authors.

## Ethics Statement

The Ethics Committee of The First Affiliated Hospital of China Medical University reviewed and approved the study protocol. Written informed consent was obtained from each participant before the study.

## Author Contributions

JJX, WMT searched the literature. HS and JJX designed the study. JZ, XJH, ZXC, QHH, JL, HBD, XXH, ZNZ, YJJ, WQG and WX collected data. JJX, ZXC and WMT analyzed data. JJX, XJH, WMT and HS interpreted data. JZ, JJX, WMT and HS wrote the Article. All authors reviewed and approved the final report. 

## Funding

This study was funded by the Mega-Projects of National Science Research (11th Five-Year Plan [2008ZX10001-001], 12th Five-Year Plan [2012ZX10001-006], and 13th Five-Year Plan [2017ZX10201101]), the National Science and Technology Major Project of China during the 13th Five-year plan period (2017ZX10201101), the Beijing Excellent Talent Plan (2018000021223ZK04), the Beijing Talent Project in the New Millennium (2020A35), the National Institutes of Health (NIAID 1R01AI114310-01), UNC-South China STD Research Training Center (FIC 1D43TW009532-01), and the UNC Center for AIDS Research (NIAID 5P30AI050410).

## Conflict of Interest

The authors declare that the research was conducted in the absence of any commercial or financial relationships that could be construed as a potential conflict of interest.
